# Bleomycin Exerts Ambivalent Antitumor Immune Effect by Triggering Both Immunogenic Cell Death and Proliferation of Regulatory T Cells

**DOI:** 10.1371/journal.pone.0065181

**Published:** 2013-06-07

**Authors:** Hélène Bugaut, Mélanie Bruchard, Hélène Berger, Valentin Derangère, Ludivine Odoul, Romain Euvrard, Sylvain Ladoire, Fanny Chalmin, Frédérique Végran, Cédric Rébé, Lionel Apetoh, François Ghiringhelli, Grégoire Mignot

**Affiliations:** 1 INSERM, U866, Dijon, France; 2 INSERM AVENIR Team, Dijon, France; 3 Faculté de Médecine, Université de Bourgogne, Dijon, France; 4 University of Bern, Institute of Pathology, Bern, Switzerland; 5 Centre Georges François Leclerc, Dijon, France; Robert Wood Johnson Medical School, United States of America

## Abstract

Bleomycin (BLM) is an anticancer drug currently used for the treatment of testis cancer and Hodgkin lymphoma. This drug triggers cancer cell death via its capacity to generate radical oxygen species (ROS). However, the putative contribution of anticancer immune responses to the efficacy of BLM has not been evaluated. We make here the observation that BLM induces immunogenic cell death. In particular, BLM is able to induce ROS-mediated reticulum stress and autophagy, which result in the surface exposure of chaperones, including calreticulin and ERp57, and liberation of HMBG1 and ATP. BLM induces anti-tumor immunity which relies on calreticulin, CD8^+^ T cells and interferon-γ. We also find that, in addition to its capacity to trigger immunogenic cell death, BLM induces expansion of Foxp3+ regulatory T (Treg) cells via its capacity to induce transforming growth factor beta (TGFβ) secretion by tumor cells. Accordingly, Treg cells or TGFβ depletion dramatically potentiates the antitumor effect of BLM. We conclude that BLM induces both anti-tumor CD8^+^ T cell response and a counteracting Treg proliferation. In the future, TGFβ or Treg inhibition during BLM treatment could greatly enhance BLM anti-tumor efficacy.

## Introduction

The aim of anti-cancer therapy is the eradication of tumor cells in the patient’s body, including the smallest metastasis or single-cell localization that could not be removed by surgery. Chemotherapy and radiotherapy are currently used to complement surgery to kill residual disease or for the treatment of metastatic disease. However, tumor cells can develop some resistance and escape cytotoxic treatment, causing tumor relapse. In this context, immunotherapy is seen as one of the ultimate anti-cancer strategies, as the immune system could reshape its actions against an evolving malignancy and eliminate tumor cells resistant to chemotherapy. Accumulating evidence shows that conventional chemotherapies, in addition to their direct cytotoxic effect, could trigger an antitumor immune response. In particular, we have shown that some chemotherapies activate natural killer (NK) cells [1], [2], some drugs target suppressive cells such as regulatory T cells (Treg) or myeloid-derived suppressor cells (MDSC) [3], [4] and some other drugs also induce a particular mode of cancer cell death called “immunogenic cell death” (ICD) [5]. ICD relies on the capacity of drugs to induce three main checkpoints. The first one is the exposure of ‘eat-me’ signals on cell surface, such as calreticulin (CRT), caused by endoplasmic reticulum (RE) stress [6], which favors cancer cell phagocytosis by dendritic cells [7]. The second is the secretion of an endogenous Toll-like receptor 4 (TLR4) ligand High-mobility group box 1 (HMGB1) [8], which is required for efficient processing of tumor antigen by dendritic cells. The third signal is the release of ATP which induces the production of interleukin (IL)-1β by dendritic cells and favors CD8^+^ T cell immune response [9].

Bleomycin (BLM) is an anti-tumor antibiotic glycopeptide produced by the bacterium Streptomyces [10]. BLM causes breaks in DNA, similar to those obtained with radiotherapy [11]. This DNA damage has been demonstrated to be mediated through induction of oxidative stress [12], [13]. BLM is indicated, in association with other cytotoxic agents, for the treatment of cancer testis and Hodgkin disease [14], [15]. The particularity of these two diseases is the high rate of cure obtained by chemotherapy. So, we proposed to test the antitumor immune response induced by BLM to determine if this mechanism could participate in the antitumor effect of bleomycin and may explain the high capacity of BLM-containing regimens to cure cancer.

In this study, we analyzed the implication of ICD, effector cells (such as CD8+ T and NK cells), as well as modulator cells (such as dendritic cells, MDSC and Treg) in BLM antitumor efficacy. We describe how BLM treatment induces an immunogenic apoptosis of tumor cells, anti-tumor CD8^+^ T cell response and the production of the tolerogenic cytokine transforming growth factor beta (TGFβ) by tumor cells which mediates regulatory T cell accumulation *in vivo*.

## Materials and Methods

### Cell Culture

MCA205 (fibrosarcoma), B16F10 (melanoma) cell lines, syngeneic of C57BL/6 mice, and CT26 (colon carcinoma) cell line, syngeneic of Balb/c mice, were cultured at 37°C in 5% CO_2_ in RPMI 1640 (Lonza) enriched with 10% fetal bovine serum, 0.4 mmol/L of sodium pyruvate, 4 mmol/L of HEPES, and antibiotics (penicillin, streptomycin, and amphotericin B). All cell lines were obtained from the American Type Culture Collection, excepted for B16-OVA, which was a kind gift from Pr Laurence Zitvogel.

### Mouse Models

#### Mice

All experiments were approved by the Université de Bourgogne’s Animal Experimental Ethics Comitee. Female C57BL/6 or Balb/c mice were purchased from the Centre d’Elevage Janvier (Le Genest St. Isle, France) and used between 6 and 10 weeks of age. OT-I transgenic mice (C57BL/6-Tg(TCRαTCRβ)1100mjb) were provided by C.Borg (INSERM U645, Besançon, France). Foxp3-EGFP [16] and CD45.1 syngeneic mice were from CDTA (UMR 7355 CNRS, Orléans, France).

#### Tumor models

CT26 tumor cells were injected into the right flank of Balb/c mice (1.10^6^ cells). Tumor size was monitored three times a week with a caliper. In some experiments, 1.10^6^ cells were injected intraperitoneally to induce peritoneal carcinosis.

#### Treatments

Mice treated by BLM received 5 intraperitoneal injections (20 mg/kg) every other day, starting when tumor size reached approximately 25 mm^2^. Mice receiving LPS (InVivoGen, Toulouse, France) were treated intraperitoneally (40 µg) the day before sacrifice. Mice treated with IL-2 received 2 intraperitoneal injections per day (1.10^5^ UI, Chiron), starting two days before sacrifice. Anti-CD8 (2.43), anti-CD4 (GK1.5), anti-CD25 (PC-61.5.3) and anti-TGFβ (1D11) antibodies were bought from BioXcell (West Lebanon, NH, USA) and used to eliminate CD8^+^, CD4^+^, CD25^+^ Treg lymphocytes or TGFβ *in vivo*, respectively. Injections were performed intraperitoneally, 150 µg per mouse 12 hours before and 12 hours after first BLM injection, and then 100 µg per mouse twice a week.

#### Vaccination

B16F10 cells expressing chicken ovalbumin (B16-OVA) were treated for 24 hours using the indicated drugs, and were then injected (5.10^5^ cells) into the footpad of C57BL/6 mice. Positive control was 1 mg of ovalbumin (Calbiochem, Merck France) protein plus 10 µg CpG ODN 1668 (InVivoGen). Five days later, draining lymph nodes were harvested, mechanically disrupted and restimulated for 72 hours with PBS, OVA (1 g/L) or anti-CD3 (2 µg/mL, 145.2C11, BioXcell) plus anti-CD28 (2 µg/mL, PV1, BioXcell). Then supernatants were collected and IFNγ concentration was determined using ELISA (BD biosciences, le Pont de Claix, France).

### *In vitro* Treatments

Tumor cells were treated for 24 hours with 30 µM Mitomycin C, 0.25 µM doxorubicin, 30 mM N-acetyl-cystein, or 15 µg/mL BLM for CT26 and MCA205 cells, 150 µg/mL BLM for B16F10.

#### Suppression assays

We used OT-I suppression assay [4], [17], [18], adapted as follow. Five days after PBS or BLM treatment, spleens from tumor bearing mice were harvested and mechanically disrupted. MDSC were labeled as PE-Cy7 Gr1^+^ cells and isolated with anti-PE-Cy7 magnetic beads (Miltenyi). CD4^+^ lymphocytes were purified using MACS CD4^+^ T cells isolation kit II (Miltenyi, Paris, France), and then Treg were purified among CD4^+^ lymphocytes, using CD25 microbeads (Miltenyi): CD4^+^ CD25^+^ were labelled Treg and CD4^+^ CD25^−^ cells were used as Tconv. Separately, OT-I transgenic mice were sacrificed and lymph nodes and spleens harvested. CD8^+^ lymphocytes were then purified using MACS CD8^+^ T cells isolation kit II. CD8^+^ OT-I lymphocytes were cocultured for 72 hours with SIINFEKL peptide (10 µg/mL, Tebu) and purified Treg or Tconv (1∶1 cells) from tumor bearing mice. IFNγ concentration in the supernatants was determined by ELISA (BD bioscience).

#### Cell transfer

CD45.2 Foxp3-EGFP mice were sacrificed then lymph nodes and spleens were harvested. These organs were mechanically disrupted and the single cell suspension was enriched in CD4^+^ lymphocytes using MACS CD4^+^ T cells isolation kit II (Miltenyi). Then CD4^+^ GFP^−^ CD62L^+^ cells (naïve CD4^+^ T lymphocytes) and CD4^+^ GFP^+^ cells (Treg) were sorted using FACS Aria III (BD). In vivo, sorted naive T cells and Treg cells were injected intraveinously into CD45.1 C57BL/6 CT26 tumor bearing mice. CD45.1 mice which had received Treg were then treated with PBS, BLM or IL-2. CD45.1 mice which had received naïve CD4^+^ T cells were treated with PBS or BLM. The day before sacrifice, CD45.1 mice which had received Treg were treated by EdU (70 mg/kg, intraperitoneal). Five days after BLM treatment, all CD45.1 mice were sacrificed and spleens and tumors harvested. Organs were mechanically disrupted, and a CD4 CD45.2 Foxp3 EdU (Click-iT EdU kit, Invitrogen, Saint Aubin, France) staining was performed. Cells were then analysed by flow cytometry. In vitro, Treg were cultured with supernatant from PBS-or BLM-treated CT26 cells in presence of anti-CD3 and anti-CD28 antibodies. Three days later, cells were incubated 3 h with BrdU, then handled with BD BrdU Flow Kit, according to the manufacturer’s protocol and FACS-analysed.

### Flow Cytometry

Five days after last BLM treatment, tumor bearing mice were sacrificed and organs were harvested, and mechanically disrupted. The cell suspensions from spleen or tumor were separated with lymphocyte separation medium (Eurobio) to isolate live mononuclear cells. Cells were stained with the antibodies described below, according to the manufacturer’s protocol. Foxp3 staining was performed using mouse regulatory T cell staining kit (eBiosciences). Dead cells were excluded by DAPI labeling (1 µg/mL). Following antibodies were from BD Biosciences : APC-H7 anti-CD19, PerCP anti-CD3, V500 anti-CD4, V450 anti-CD8, PE anti-DX5, FITC anti-CD11c, PE anti-Ly6G, PerCP anti-CD4, APC anti-CD3, FITC anti-CD40, PE anti-CD86, Alexa700 anti-CD62L, V450 anti-CD45.2, APC anti-CD11c,; the following antibodies were from BioLegend: Alexa700 anti-F4/80, APC anti-LAP; the following antibodies were from eBiosciences: APC anti-CD11b, PerCP-Cy5.5 anti-Ly6C, eFluor450 anti-Foxp3, Alexa700 anti-CHM-II. Other antibodies were also used: Alexa647 azide (Invitrogen), anti-CRT rabbit whole serum (Abcam), anti-ERp57 rabbit whole serum (Abcam), Alexa 680 anti-rabbit (Invitrogen). Data were acquired with LSR II (BD), and analysed with FlowJo (Tristar). Cell sorting was done with a BD ARIA III. Cell populations were defined as below: B lymphocytes: CD19^+^; CD4^+^ and CD8^+^ T lymphocytes: CD3^+^ CD4^+^ or CD3^+^ CD8^+^, respectively; NK lymphocytes: CD19^−^ CD3^−^ DX5^+^; dendritic cells: CD19^−^ CD3^−^ CD11c high; monocytes/macrophages: CD11b^+^ F4/80^+^; monocytic or granulocytic MDSCs: CD11b^+^ Ly6C^+^ or CD11b^+^ Ly6G^+^, respectively; Treg: CD3^+^ CD4^+^ Foxp3^+^; dendritic cells maturation: fluorescence intensity of CD40 and CD86 labelling in live CD11c high MHC-II^+^ cells.

### Autophagy Detection

Cyto-ID kit (Enzo) was used according to the manufacturer’s protocol for flow cytometry and immunofluoresence. Anti-LC3 (clone 5F10, Nanotools) was used on cells fixed and permabilized with BD fix/perm kit and revealed with alexa 488 anti-mouse (InVitroGen).

### ROS Detection

Cells were incubated 2 hours with the indicated drugs, washed, then incubated with 100 µM DCFH_2_/DA (Sigma) for 50 min at 37°C, and analysed by flow cytometry.

### Immunoblot Analysis

Samples were lysed in lysis buffer containing 1% SDS (Promega), 10 mM Tris HCl pH 7.5, 1 mM NaF (Sigma), 1 mM EDTA (Promega), 2 mM sodium orthovanadate (Sigma) and antiprotease cocktail (Roche, Mannheim, Germany). The protein concentration for each sample was determined by the DC protein assay according to the manufacturer’s protocol (Bio-rad, Marnes-la-Coquette, France). After immunoblotting, immunochemical detection was revealed with an appropriate horseradish peroxidase-labeled secondary antibody (Jackson ImmunoResearch). Specific bands were visualized using a chemiluminescence detection system (Santa Cruz, Heidelberg, Germany), according to the manufacturer’s protocol. Controls of equal loading of proteins were performed with anti-actin antibody. The following antibodies were used: monoclonal rabbit anti-p-eIF2α (Cell Signaling), monoclonal rabbit anti-eIF2α (Cell Signaling), monoclonal mouse anti-β-actin (Sigma).

### Extracellular ATP Concentration

Supernatants were centrifugated twice (425 *g*, 3 min, room temperature) to eliminate cellular debris. Then ATP concentration was determined with the Cell Titer Glo Viability Assay Kit (Promega), according to the manufacturer’s protocol.

### ELISA

The following kits were used according to the manufacturer’s protocol: HMGB1 detection kit (Chondrex), IFNγ ELISA kit (BD), TGFβ1 Ready-SET-Go ELISA kit (eBiosciences).

### Primers and PCR

Nucleic acid purification was performed using Trizol (Invitrogen), according to the manufacturer’s protocol. The M-MLV Reverse Transcriptase kit (Invitrogen) was used according to the manufacturer’s protocol to perform reverse transcription. RT-qPCR was performed using the *Power* SYBR® Green PCR Master Mix kit (Applied Biosystems), according to the manufacturer’s protocol with a 7500 Fast Real Time PCR system (Applied Biosystems). The following primers were used: *b-actin* (internal control): forward 5′-ATGGAGGGGAATACAGCCC-3′, reverse 5′-TTCTTTGCAGCTCCTTCGTT-3′; *Tgfb1*: forward 5′-CAACCCAGGTCCTTCCTAAA-3′, reverse 5′-GGAGAGCCCTGGATACCAAC-3′.

### shRNA Transduction

CT26 cells were transduced with MISSION Lentiviral Transduction Particles (Sigma-Aldrich) to silence CRT or with negative control particles according to the manufacturer’s instructions.

### Statistical Analysis

Data were analysed with GraphPad Prism 5 Software. Comparisons between control and test conditions were performed with student t test or Mann&whitney test; group comparison were performed with Kruskal-Wallis test followed by Dunn’s post-test. Tumor growth comparisons were performed by two-way ANOVA method.

## Results

### BLM Induces Stigmata of Immunogenic Cell Death

**Figure 1:**
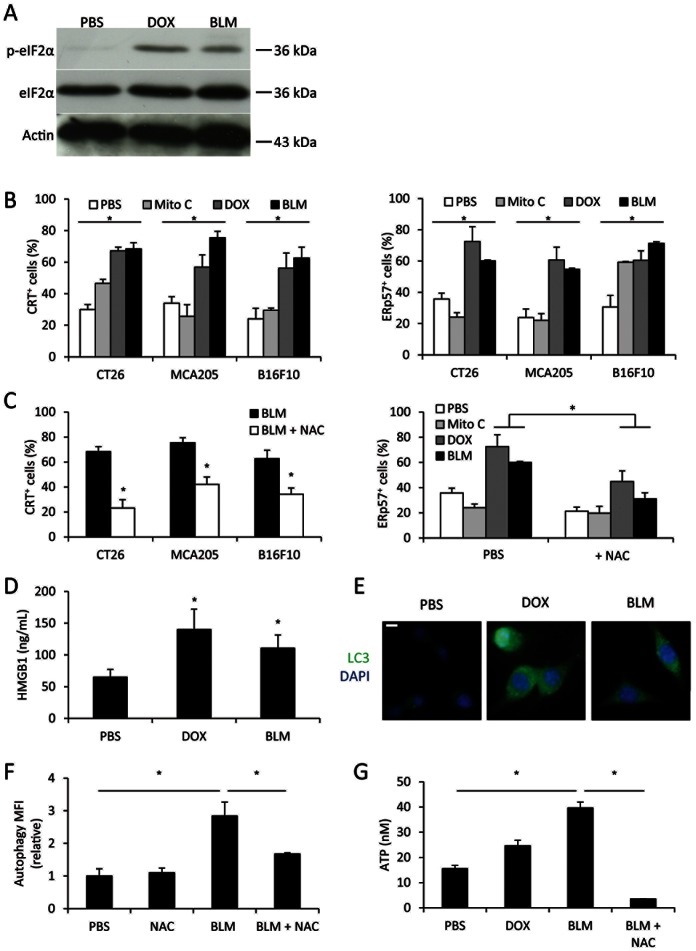
BLM treatment triggers characteristic events of immunogenic cell death. A: Western blotting analysis of phosphorylated, total eIF2α and β-actin in CT26 treated 24 h with the indicated drugs. B: indicated tumor cells were treated *in vitro* with different chemotherapeutic drugs for 24 h, then CRT (left panel) or ERp57 (right panel) exposure was detected by flow cytometry. C: tumor cells were treated with BLM, with or without N-acetylcystein (NAC) for 24 h, then CRT (left panel) or ERp57 (right panel) exposure was detected by flow cytometry. C: HMGB1 was titrated with ELISA method in supernatant of CT26 cells treated 24 h with indicated drugs. E: CT26 cells were cultured on glass slides and treated with indicated drugs for 24 h. The cells were then submitted to anti-LC3 (green) and DAPI (blue) labeling and observed under an epifluorescence microscope. F: CT26 cells were treated with indicated drugs for 24 h, then stained using Cyto-ID autophagy detection kit and analysed by flow cytometry. G: ATP was titrated in supernatant of CT26 treated with indicated drugs for 24 h. All data presented are representative of one out of three experiments. B-D, E and F show mean +/− SEM.

BLM antitumor effect is due to its capacity to induce radical oxygen species (ROS) production [19], [20]. Because ROS generation could lead to reticulum stress [21], we hypothesized that BLM could be able to cause ROS-dependent ER stress and the induction of ICD. We found that BLM, like doxorubicin (DOX), an anthracycline and immunogenic drug [7], triggers eIF2α phosphorylation, a marker of reticulum stress in CT26 cells (Figure 1a). Chemotherapy-induced reticulum stress could trigger calreticulin (CRT) and the endoplasmic reticulum chaperone ERp57 translocation at the cell surface [22], [23]. *In vitro*, BLM induced translocation of CRT and ERp57 onto tumor cell plasma membrane in three mouse cancer models (Figure 1b). As expected, BLM effects are comparable to DOX effects, whereas mitomycin C (Mito C), a non-immunogenic drug [7], did not induce CRT or ERp57 exposure, (Figure 1b). Importantly, we showed that these translocations are aborted by using N-acetylcystein (NAC) (Figure 1c), a ROS inhibitor (Figure S1). The second determinant of ICD is the capacity of chemotherapy to induce the release of HMGB1 which triggers TLR4 on antigen presenting cells [8]. Using ELISA, we observed that BLM induces similar release of HMGB1 as doxorubicin (Figure 1d). The third element of ICD is the capacity of the chemotherapeutic agent to trigger autophagy and subsequent ATP secretion which activates dendritic cells via P2RX7 [9], [24]. We observed that BLM induced, like DOX, LC3 puncta in immunofluoresence and Cyto-ID labeling in flow cytometry, two markers of autophagy vacuoles (Figure 1e, 1f and Figure S2). Of note, mitomycin C was unable to induce these signs of autophagy (Figure S2). Similarly to DOX, BLM also induced ATP release from CT26 cells (Figure 1g). The ROS inhibitor NAC also blunted both autophagy and ATP release (Figure 1f and 1g).

Together, these data demonstrate that BLM triggers all characteristic events of immunogenic cell death [7]–[9] in a ROS-dependent manner.

### BLM-induced Immunogenic Cell Death Drives CD8^+^ Dependent Antitumor Immunity

**Figure 2:**
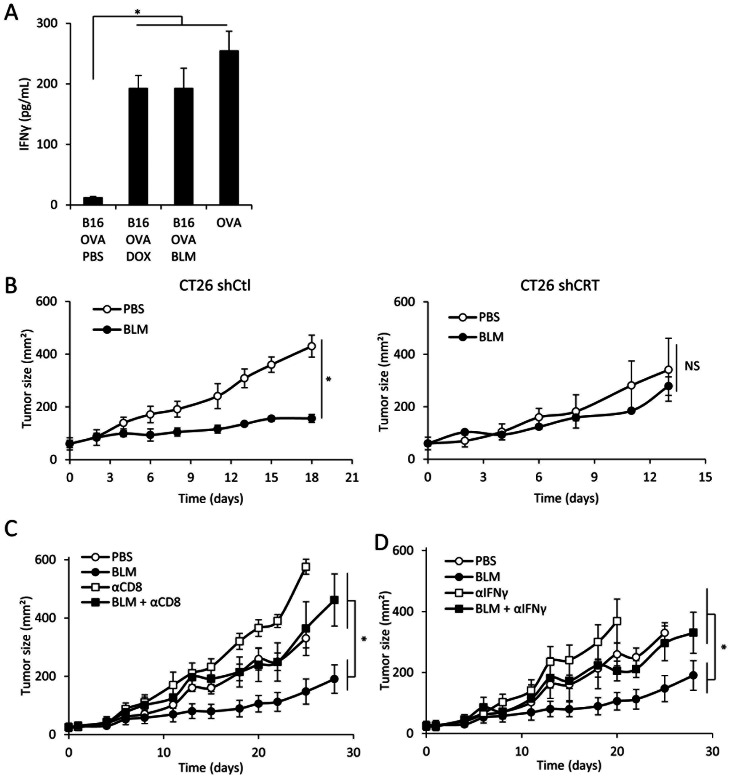
BLM in vivo antitumor effect through immune-based mechanisms. A: Footpad injection of different vaccines was performed: PBS-, DOX- or BLM-treated B16-OVA, or ovalbumin protein. 5 days later, the popliteal lymph nodes were harvested and cells were rechallenged with OVA peptide SIINFEKL. After 3 days of culture, IFNγ was titrated in the supernatant using ELISA method. B: shControl CT26 (left panel) or shCRT CT26 (right panel) were injected in the flank of mice. When the tumor reached about 25 mm^2^, mice were treated with PBS or BLM and tumor growth was monitored with a caliper over time. C and D: CT26 cells were injected in the flank of mice. When the tumor reached about 25 mm^2^, mice were treated with PBS or BLM. Some of the mice received injection of isotype control or depleting anti-CD8 (C) or anti-IFNγ (D). We monitored tumor growth with a caliper over time. All data presented are representative of one out of two experiments. Graphs show mean +/− SEM.

To determine whether BLM-treated tumor cells induced antitumor immune response *in vivo*, we vaccinated naïve mice by injecting B16-OVA cells treated with PBS, BLM or DOX in the footpad. We observed that BLM- and DOX- treated cells are able to induce ovalbumin-dependent IFNγ production by T cells from the draining lymph node (Figure 2a). We also performed *in vivo* vaccination with control or CRT-knocked down BLM-treated dying tumor cells, followed by a challenge with live tumor cells. We could observe that the BLM-treated control CT26 cells were able to induce protective immunity (100% protection, n = 10), in contrast to mitomycin-treated CT26 (20% protection, n = 10) and CRT-invalidated BLM-treated cells (30% protection, n = 10). Furthermore, in the setting of growing s/c CT26 tumors, we observed that 5 injections of BLM delayed the growth of control shRNA transfected CT26 cells but had no effect on CRT shRNA transfected CT26 cells (Figure 2b). Finally, IFNγ or CD8 depletion completely prevents BLM antitumor effect *in vivo* (Figure 2c and 2d). Together, these data strongly suggest that BLM exerts a calreticulin-, IFNγ- and CD8-dependent antitumor effect.

### BLM Shows No Effect on Innate Immune Cells

In addition to its capacity to elicit ICD of tumor cells, chemotherapy could harness the host immune system by inducing dendritic cell maturation [25], NK cell activation [1] or by eliminating immunosuppressive cells such as Treg and MDSC [3], [4], [26].

**Figure 3:**
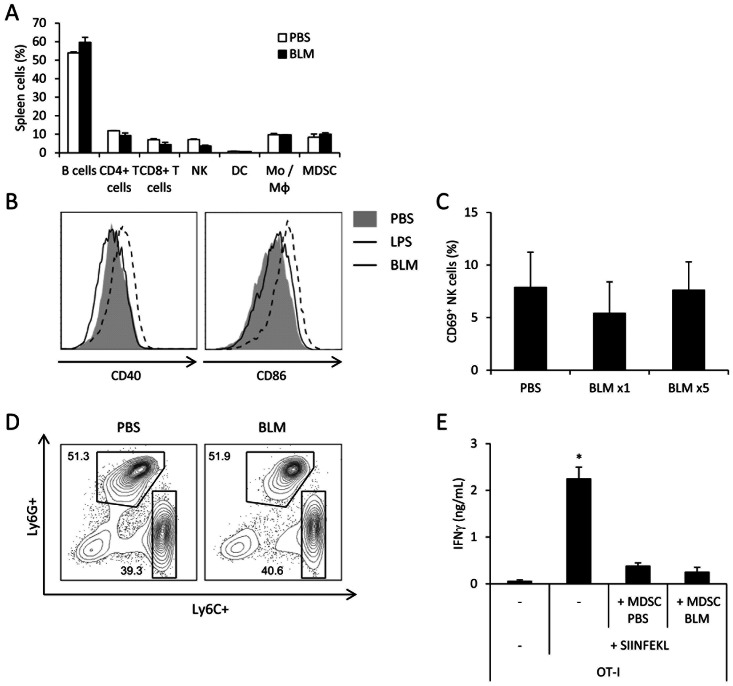
BLM has no obvious effect on innate immune cells. A: Spleens from CT26 tumor-bearing mice, treated with PBS or BLM, were harvested and the cells were analyzed through flow cytometry. The graph shows proportion of different splenocyte populations identified by immunostaining. B: Mice received a single injection of PBS, LPS or BLM. One day later, spleens were collected and the maturation status of dendritic cells defined as CD11c^hi^ and MHC-II^+^ was assessed by flow cytometry, with CD40 (left panel) and CD86 (right panel) staining. C: We injected CT26 cells in the thigh of mice. When the tumor reached about 25 mm^2^, we treated them with PBS, single or five BLM injections. One day after last injection, inguineal lymph nodes were collected and NK cell activation was analysed through CD69 immunostaining. D: Same as A with further identification of MDSC sub-populations with Ly6G and Ly6C immunostaining. The cells are gated as CD11b^hi^ among the live cells. E: MDSC from PBS- or BLM- treated CT26-bearing mice were isolated from spleen and co-cultured with OT-I in presence of SIINFEKL. After 3 days of culture, IFNγ was titrated in the supernatant using ELISA method. All data presented are representative of one out of three experiments. Graphs show mean +/− SEM.

To address these possibilities in the BLM treatment, we have first assessed if BLM injection did significantly modify the number of B cells, CD4+ T cells, CD8+ T cells, NK cells, dendritic cells, monocytes/macrophages or MDSC in spleen of tumor- bearing mice (Figure 3a). We observed that BLM treatment did not change the proportion of all the cell types analyzed. We further tested whether BLM promoted the maturation of antigen presenting cells by testing the effect of a single systemic injection of BLM or lipopolysaccharide (LPS) on the maturation pattern of splenic dendritic cells (DC). While LPS induced as expected a massive up-regulation of CD86 and CD40, BLM administration did not affect the expression of these markers, ruling out any direct DC-activating properties of BLM (Figure 3b).

We also checked NK cell activation in tumor draining lymph nodes. We did not observe any NK activation, assessed by CD69 expression, after either PBS, one or five BLM injections (Figure 3c).

Finally, we evaluated the frequency of MDSC in the spleen of BLM-treated tumor-bearing mice. We found that BLM did not affect the number of myeloid or granulocytic MDSC subsets (Figure 3d) and did not affect MDSC immunosuppressive function, as assessed by MDSC capacity to blunt OT-I capacity to produce IFNγ (Figure 3e).

Altogether, these data suggest that BLM does not affect innate immune cells such as dendritic cells, NK cells or MDSC.

### BLM Mediates Regulatory T Cell Proliferation via Tumor Cell-derived TGFβ

**Figure 4:**
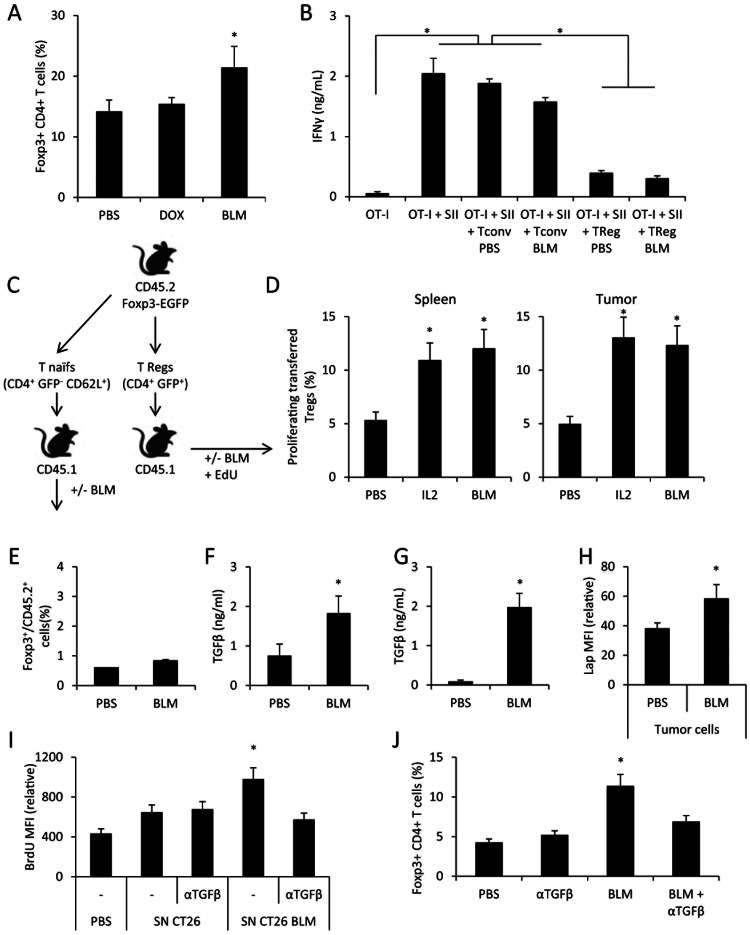
BLM induces in vivo Treg accumulation throught TGFb production. A: Spleens from CT26 tumor-bearing mice, treated with PBS or BLM, were harvested and the cells were analyzed through flow cytometry for Foxp3^+^CD4^+^ Treg detection. B: Treg from PBS- or BLM- treated CT26-bearing mice were isolated from spleen and co-cultured with OT-I in presence of SIINFEKL (SII). After 3 days of culture, IFNγ was titrated in the supernatant using ELISA method. C: Schematic representation for D and E panel experiments. D: GFP^+^ CD4^+^ cells were sorted from CD45.2 FOXP3-EGFP mice, then injected i.v. in CD45.1 mice bearing CT26 tumor. The mice then received PBS, IL-2 or BLM treatment. All mice received EdU injection. One day after treatment, spleens and tumors were collected and the proliferation status of transferred cells was assessed by revealing EdU by flow cytometry. E: CD4^+^ CD62L^+^ GFP^−^ naive T cells were sorted from CD45.2 FOXP3-EGFP mice, and injected i.v. in CD45.1 mice bearing CT26 tumor. The mice then received PBS or BLM treatment. Spleens were harvested and the cells were analyzed through flow cytometry for Treg detection. F: Mice were injected with 1.10^6^ CT26 cells i.p. Ten days later mice received PBS or BLM injection. The following day ascites were collected, and TGFβ was assessed using ELISA method. G: CT26 cells were treated with PBS or BLM *in vitro* for 24 h, then the supernatant was collected and assessed for TGFβ using ELISA method. H: Mice bearing CT26 tumor cells were treated with PBS or BLM. The day after, CT26 tumors were collected and tumor cells were separated from the tumor infiltrating lymphocytes. Tumor cells were stained for LAP and analyzed by flow cytometry. I: GFP^+^ CD4^+^ cells were sorted from CD45.2 FOXP3-EGFP mice, then cultured *in vitro* under TCR-stimulating conditions. We added culture supernatant of CT26 treated for 24 h with PBS of BLM. In some wells, blocking anti-TGFβ antibody was added. After three days of culture, the cells were incubated with BrdU for 3 h, and then proceeded to BrdU detection by flow cytometry. J: Same as A, and mice received injection of blocking anti-TGFβ antibody. All data presented are representative of one out of two (panels B, D, E, G and I) or three experiments (panels A, F, H and J). Graphs show mean +/− SEM.

While we detected no change in most immune cell populations (Figure 3a), we observed an accumulation of Foxp3^+^ Treg in lymphoid organs of mice treated with BLM and bearing CT26 (Figure 4a) or EL4 (not shown) tumors.We then determined the biological function of these Treg. We sorted CD4^+^ CD25^+^ (Treg) and CD4^+^ CD25^−^ (conventional T) T cell subsets from the spleen from PBS- or BLM-treated tumor-bearing mice, and tested their capacity to blunt IFNγ production by OT-I cells in an *in vitro* assay. We observed as expected that conventional T cells were unable to blunt OT-I capacity to produce IFNγ. In contrast, Treg from untreated or BLM-treated mice had strong and similar capacities to blunt OT-I IFNγ production (Figure 4b).

Higher Treg presence could be due to differentiation of naive T cells into Treg or expansion of pre-existing Treg. To test these hypotheses, we transferred CD45.2 CD4^+^ GFP^+^ (Treg) or CD45.2 CD4^+^ GFP^−^ CD62L^+^ (naïve T cells) from Foxp3-EGFP mice into CD45.1 tumor bearing mice (Figure 4c). We observed that BLM induces proliferation of Treg at similar level to IL-2 injection, a known Treg inducer (Figure 4d) [27], [28]. In contrast, BLM was unable to induce differentiation of naive T cells into Treg (Figure4e).

Treg cell proliferation is known to be driven by IL-2 production [28], [29] or TGFβ [30], [31]. We tested the presence of IL-2 and TGFβ in ascites of a CT26 peritoneal carcinosis model. While we could not detect any IL-2 in ascites from PBS or BLM-treated mice (not shown), we observed an induction of TGFβproduction in the ascites of BLM-treated animals (Figure4f). *In vitro*, we also observed that BLM treatment induced TGFβ production by CT26 both at the mRNA (Figure S3) and protein level (Figure4g). Similarly, we observed that BLM could induce *tgfb* mRNA expression in B16F10, EL4 and MCA205 cells (not shown).

To validate that TGFβ was also produced *in vivo* by tumor cells, we determined the expression of latency-associated protein (LAP), a protein associated with TGFβ, in cancer and immune cells. Only tumor cells isolated from subcutaneous tumor showed increased LAP expression after BLM treatment (Figure 4h and Figure S4).

To test if this TGFβ production by tumor cells may be responsible for Treg accumulation in BLM-treated mice, we sorted CD4^+^ GFP^+^ from Foxp3-EGFP mice and stimulated them with T cell receptor (TCR) triggering. In some conditions, supernatant from PBS- or BLM-treated CT26 cells was added, with or without anti-TGFβ blocking antibody. After 72 h of stimulation, the proliferation of Treg was assessed by a BrdU incorporation assay. We could observe that supernatant from BLM-treated CT26 induces proliferation of Treg, and that this effect is dependent on TGFβ (Figure 4i). *In vivo*, we also observed that inhibition of TGFβ with a blocking anti-TGFβ antibody prevents the BLM-induced accumulation of Treg in tumor-bearing mice (Figure 4j).

Taken together, these data strongly suggest that BLM induces Treg accumulation through enhancement of tumor-produced TGFβ.

### TGFβ or Regulatory T cell Depletion Potentiate the Antitumor Effects of BLM

**Figure 5:**
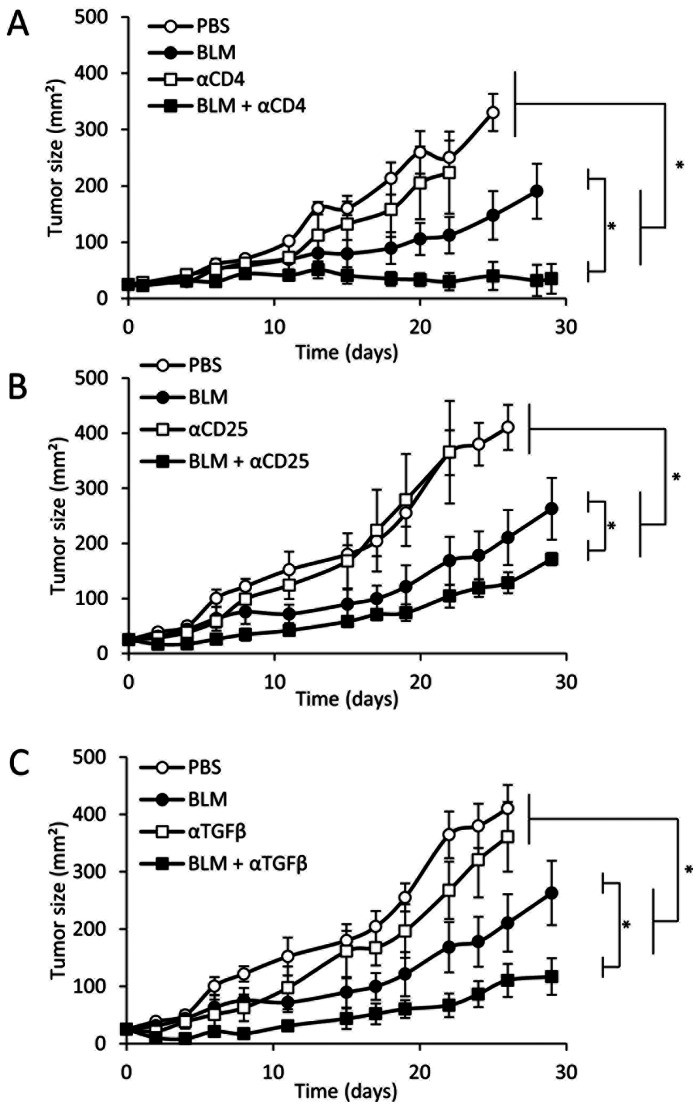
BLM anti-tumor effect is enhanced by Treg or TGFβ depletion. Five hundred thousand CT26 cells were injected in the flank of mice. When the tumor reached about 25 mm^2^, mice were treated with PBS or BLM. Some of the mice received injections of isotype control antibody or depleting anti-CD4 (A), anti-CD25 (B) or blocking anti-TFGβ (C) antibodies. Tumor growth was monitored with a caliper over time. All data presented are representative of one out of two experiments. Graphs show mean +/− SEM.

To assess the therapeutic relevance of these observations, we tested the effect of Treg depletion on the antitumor effect of BLM. In the colon carcinoma model CT26. We first observed that the antitumor effect of BLM was drastically improved by CD4^+^ T cell depletion (Figure 5a). To validate that the antitumor effect of CD4 depletion is mediated essentially by Treg, we tested the effect of an anti-CD25, which is a membranous marker for mouse Treg, depleting antibody. We observed that CD25 depletion also enhanced the antitumor efficacy of BLM (Figure 5b).

We demonstrated above (Figure4) that BLM induces TGFβ production by tumor cells and Treg expansion in tumor bearing mice. To determine if this phenomenon impacts on BLM antitumor effect, we treated tumor-bearing mice with either BLM, anti-TGFβ or both. While the anti-TGFβ antibody did not significantly impact on tumor growth, TGFβ depletion enhanced the antitumor efficacy of BLM (Figure 5c).

Taken together, these data highlight that BLM treatment causes TGFβ-dependent proliferation of Treg, which decreases its anti-tumor efficacy.

## Discussion

Our study reveals that BLM can be counted among the immunogenic anticancer drugs which trigger ICD along with oxaliplatin and anthracyclines [7], [8], [32]. Here, by studying the immune-based effects of BLM anti-cancer treatment, we show that BLM is capable to cause ER and oxidative stress, CRT exposure, HMGB1 release, autophagy and ATP release, providing all the elements required to induce an ICD, leading to IFNγ and CD8^+^ T cell response against tumor cells. Importantly, BLM has a two-edged effect, because BLM also induces the production of TGFβ by tumor cells and consequent Treg accumulation which limits its anti-tumor efficacy. These data demonstrate that BLM may have ambivalent immune effects which limit the antitumor efficacy of this anticancer agent. This is similar to our recent conclusions on the immune effects of 5-fluorouracile (5 FU) on tumor immunity. We indeed described that 5 FU induces MDSC death, which restores CD8^+^ T cell antitumor immunity and causes initial efficacy of chemotherapy [4]. But while MDSC die, they release IL-1β, which induces accumulation of deleterious Th17 cells, promoting later tumor relapse [33]. Together, these observations about 5 FU and BLM underscore the importance to perform a systematic analysis of the immune effect of anticancer agents on the different aspects of the immune response.

Besides, cardiac glycosides, such as digoxin, were recently identified as strong immunogenic cell death inducers, using a high throughput strategy [34]. In this study, it was shown in rodent models that treatment of tumor cells with digoxin can enhance the effects of non-immunogenic cytotoxic drugs, such as cisplatin [32], due to their capacity to convert non immunogenic to immunogenic cell death. However, the effect of BLM on the immune response was not detected using this strategy. So we believe that systematic analysis of immune properties of chemotherapeutic agents on each immune cell subset is complementary to high throughput strategy.

Foxp3^+^CD4^+^CD25^+^ Treg were initially defined as a subpopulation of suppressor T cells that mediate immune tolerance by suppressing autoreactive T cells [35], [36]. In human neoplasia, Treg cells accumulate in tumors, draining lymph nodes and blood, and their induction is often correlated with poor outcome both in human and rodent cancers [37]. Activation and expansion of Treg subset was determined to be dependent on IL-2 [28], [29] and TGFβ [30], [31]. BLM treatment of lung fibroblasts results in an increased transcription of *tgfb1* and increased TGFβ protein [38], [39]. This production of TGFβ by fibroblasts in the lung after BLM injection is responsible for the induction of lung fibrosis, one of the major side-effects of BLM [40], [41]. Here we made the original observation that BLM could also trigger TGFβ production by tumor cells. We may suspect that this increase in TGFβ expression could result in immunosubversion of anticancer immune response. In particular, we showed that BLM-induced TGFβ production by tumor cells drives Treg expansion and may thus inhibit the CD8^+^ antitumor immune response induced by BLM-driven immunogenic cell death. In the B16F10 model, while either high or low BLM dosage could induce the production of TGFβ by B16F10, only high concentration of BLM could induce ICD (not shown). We may therefore raise the hypothesis that in some cases the ICD effect which only occurs with high dosage of BLM is less likely to happen *in vivo* and that the TGFβ-Treg driven induction would be predominant. We also show that Treg depletion or TGFβ inhibition significantly enhance BLM anti-tumor efficacy in our *in vivo* rodent model. Taken together with the current literature, our data suggest that TGFβ and Treg are responsible for both limited antitumor effect and major side effect of BLM [40], [41]. This may give the rationale to disrupt this phenomenon by combining BLM and Treg inhibitors, such as cyclophosphamide [3]. In the future, the association between BLM and novel drugs like anti-human CD25 (such as daclizumab) [42] or inhibitors of TGFβ signaling (such as Smad inhibitors) [43] may be beneficial for both anti-cancer efficacy and side-effect management.

In conclusion, this study supports the ambivalent immunological property of BLM by its capacity to trigger immunogenic cell death and Treg accumulation.

## Supporting Information

Figure S1 BLM treatement induces ROS production. CT26 cells were treated 24 h *in vitro* with PBS, Mitomycine C (Mito C), Doxorubicine (DOX) or bleomycin (BLM), with addition of PBS or N-acetylcystein (NAC). After 24 h of culture, production of radical species was assessed by DCFH_2_/DA detection by flow cytometry. Data presented are representative of one out of three experiments. Graphs show mean +/− SEM.
(TIF)

Figure S2 BLM treatment induces autophagy. CT26 cells were cultured on glass slide, and treated with PBS, Mito C, DOX or BLM for 24 h (scale bar: 10 µm). Upper panel: cells were fixed and permeabilized, then labeled with anti-LC3 antibody (green) and DAPI (blue). Lower panel: cells were stained using the Cyto-ID autophagy detection kit, showing autophagosomes (green) and Hoescht 33342 (blue). Data presented are representative of one out of two experiments.
(TIF)

Figure S3 BLM treatment induces *tgfb* transcription in tumor cells. CT26 cells were treated *in vitro* with PBS, BLM or DOX. After 24 h, cells were collected and their mRNA isolated, then submitted to RT-qPCR for *tgfb* expression. Results are shown standardized to *actin* expression. Data presented are representative of one out of three experiments.
(TIF)

Figure S4 BLM *in vivo* treatment induces no noticeable modification of Lap in host immune cells. Mice bearing CT26 tumor cells were treated with PBS or BLM. The day after, CT26 tumors and spleen cells were collected. The tumor cells were separated from the tumor infiltrating lymphocytes. Spleen cells and tumor-infiltrating lymphocytes were stained for LAP and analysed by flow cytometry. Data presented are representative of one out of three experiments.
(TIF)
