# Supplementary figures and images for: Bleomycin Exerts Ambivalent Antitumor Immune Effect by Triggering Both Immunogenic Cell Death and Proliferation of Regulatory T Cells
Source: PLoS One. 2013 Jun 7;8(6):e65181. doi: 10.1371/journal.pone.0065181 (PMC3676388; doi:10.1371/journal.pone.0065181)

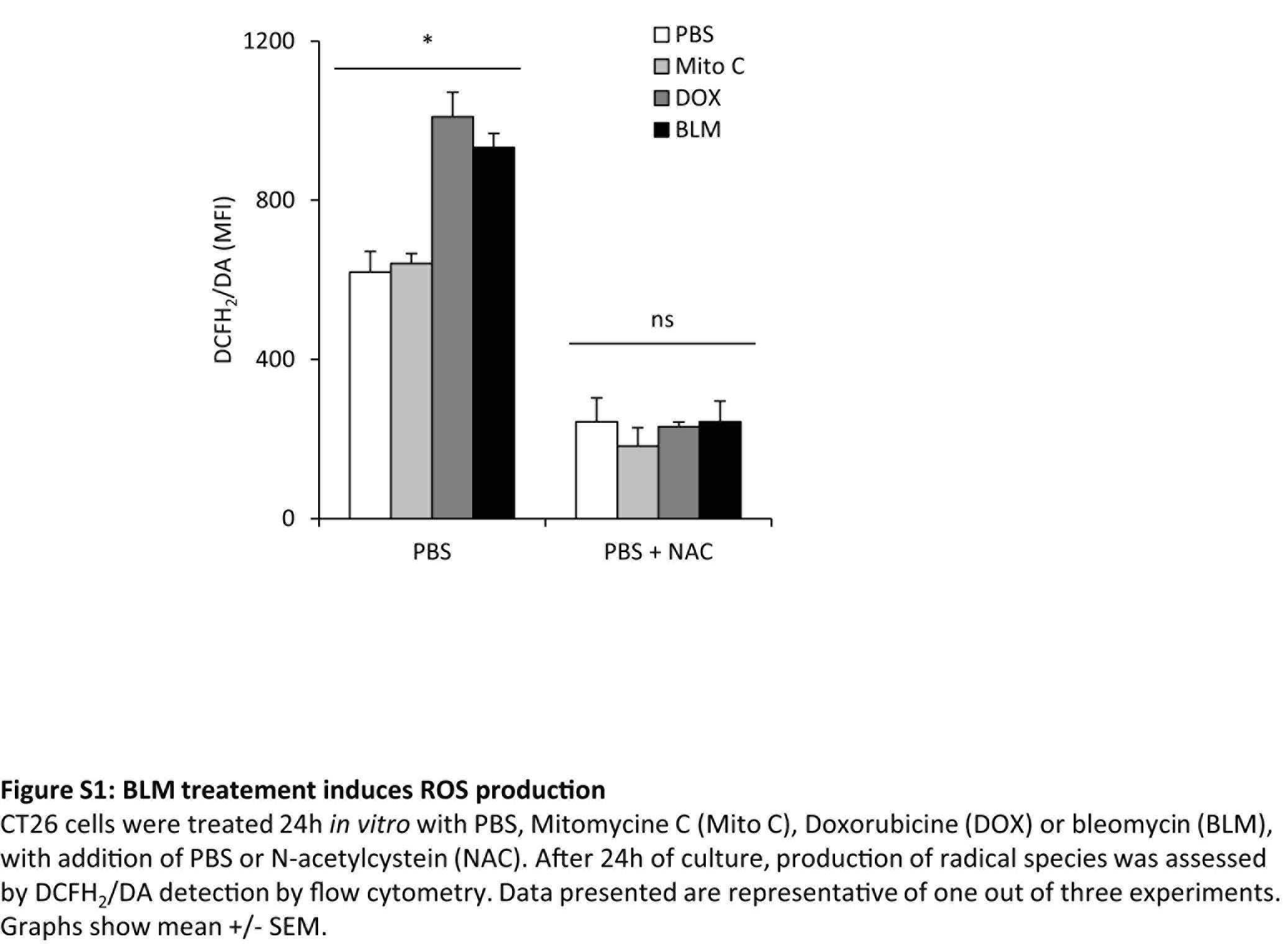

Supplement: Figure S1 — BLM treatement induces ROS production. CT26 cells were treated 24 h in vitro with PBS, Mitomycine C (Mito C), Doxorubicine (DOX) or bleomycin (BLM), with addition of PBS or N-acetylcystein (NAC). After 24 h of culture, production of radical species was assessed by DCFH2/DA detection by flow cytometry. Data presented are representative of one out of three experiments. Graphs show mean +/− SEM. (TIF) [file pone.0065181.s001.tif]

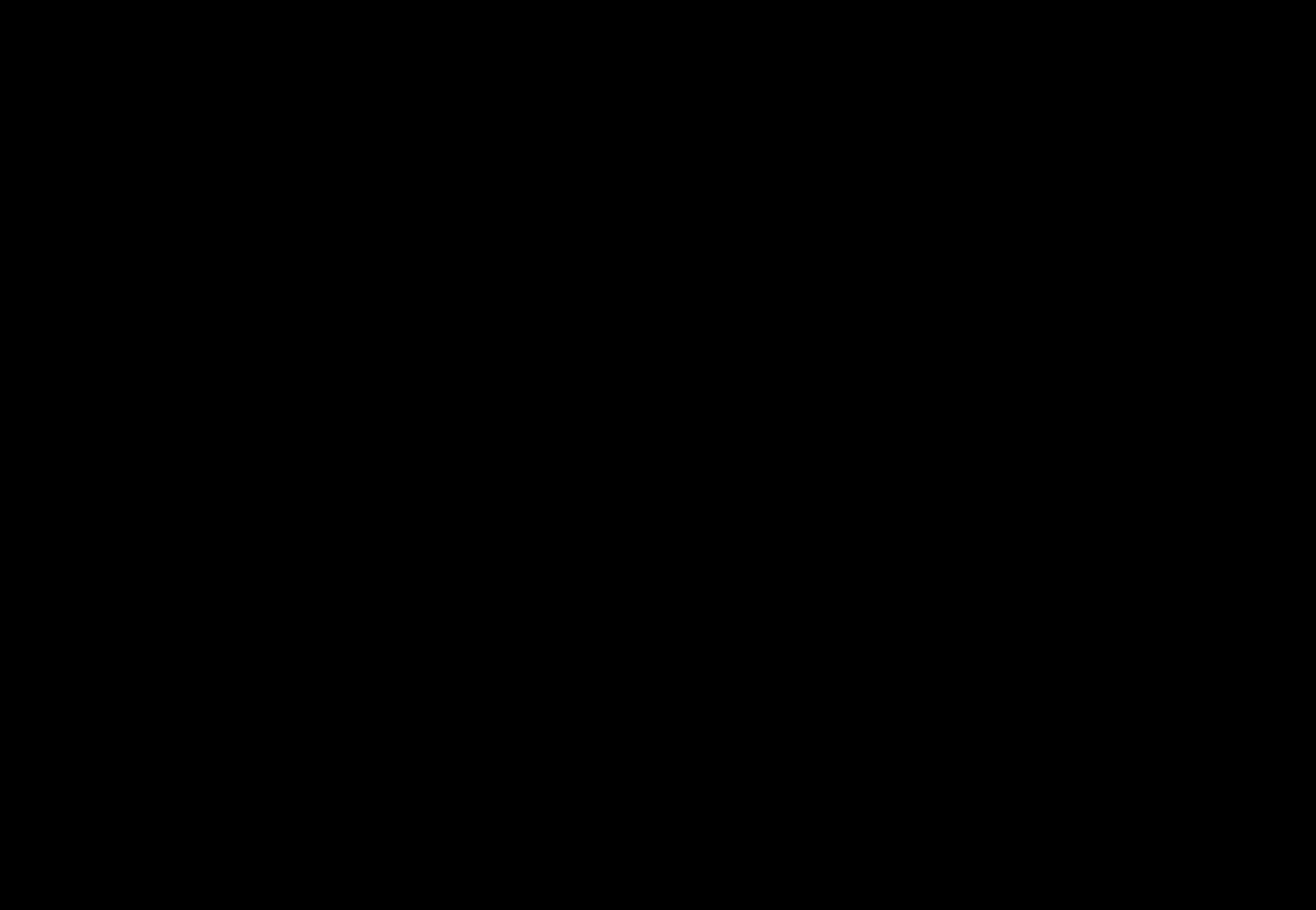

Supplement: Figure S2 — BLM treatment induces autophagy. CT26 cells were cultured on glass slide, and treated with PBS, Mito C, DOX or BLM for 24 h (scale bar: 10 µm). Upper panel: cells were fixed and permeabilized, then labeled with anti-LC3 antibody (green) and DAPI (blue). Lower panel: cells were stained using the Cyto-ID autophagy detection kit, showing autophagosomes (green) and Hoescht 33342 (blue). Data presented are representative of one out of two experiments. (TIF) [file pone.0065181.s002.tif]

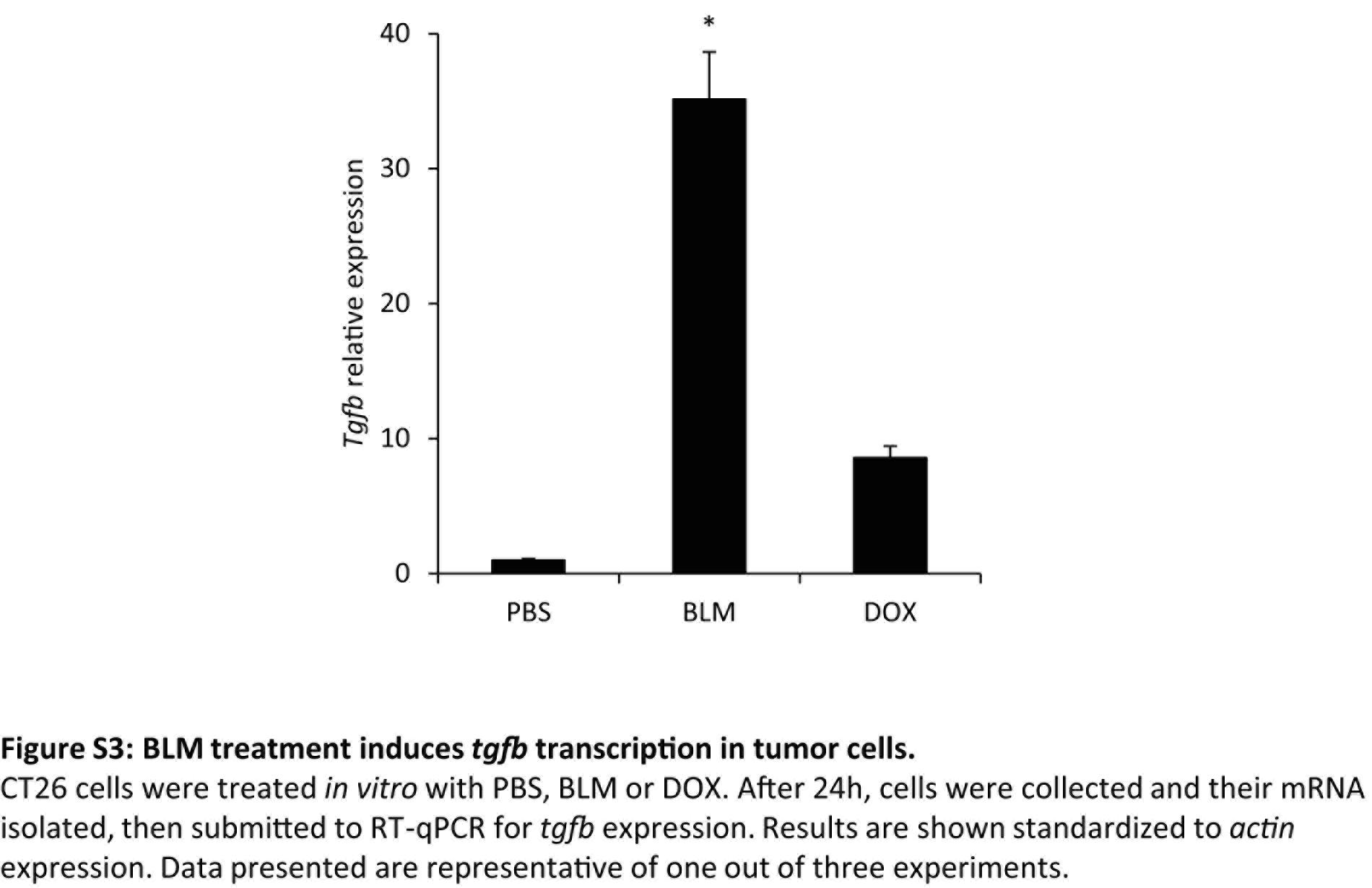

Supplement: Figure S3 — BLM treatment induces tgfb transcription in tumor cells. CT26 cells were treated in vitro with PBS, BLM or DOX. After 24 h, cells were collected and their mRNA isolated, then submitted to RT-qPCR for tgfb expression. Results are shown standardized to actin expression. Data presented are representative of one out of three experiments. (TIF) [file pone.0065181.s003.tif]

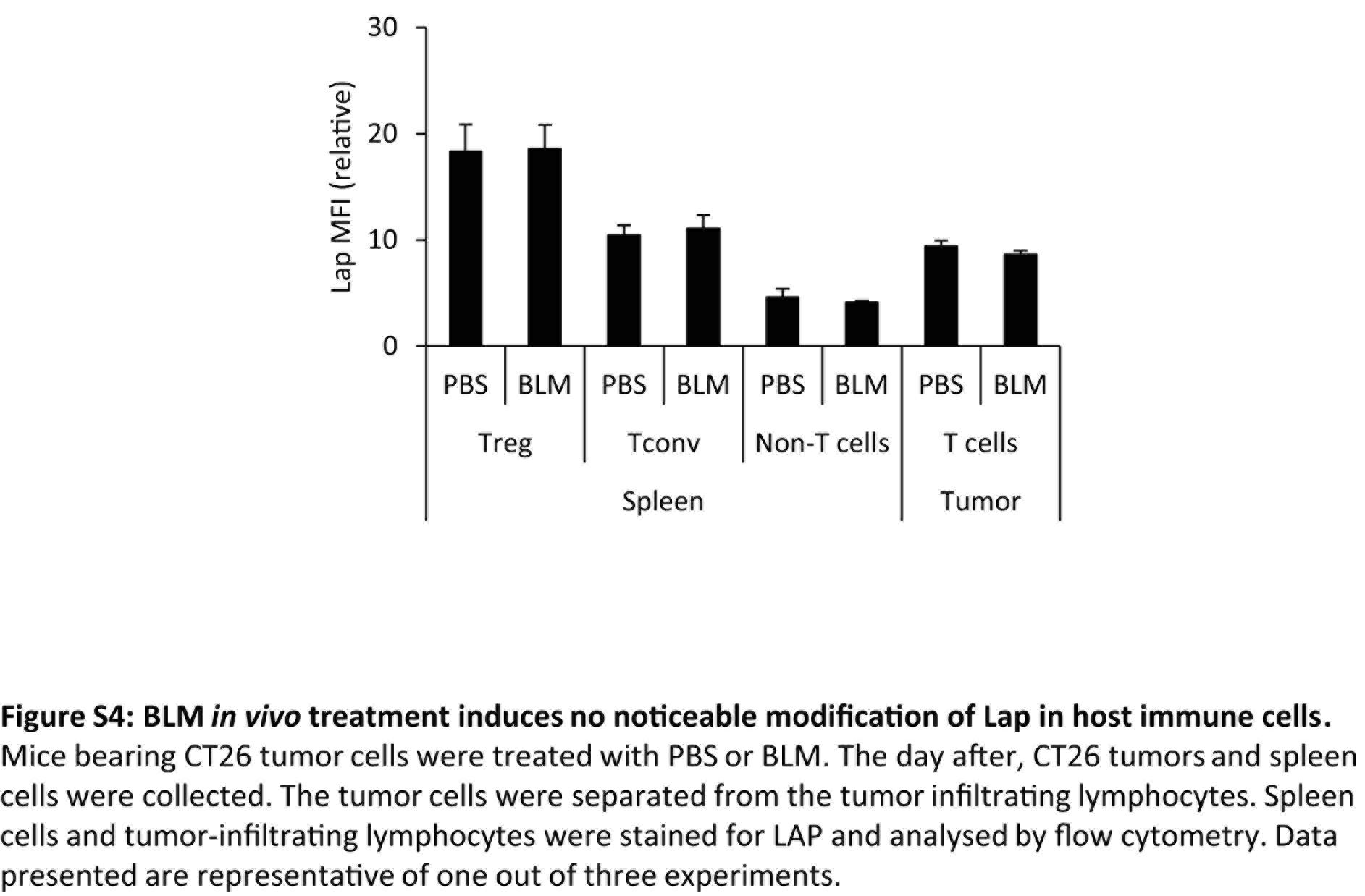

Supplement: Figure S4 — BLM in vivo treatment induces no noticeable modification of Lap in host immune cells. Mice bearing CT26 tumor cells were treated with PBS or BLM. The day after, CT26 tumors and spleen cells were collected. The tumor cells were separated from the tumor infiltrating lymphocytes. Spleen cells and tumor-infiltrating lymphocytes were stained for LAP and analysed by flow cytometry. Data presented are representative of one out of three experiments. (TIF) [file pone.0065181.s004.tif]
